# Primary Cell Cultures in Neurobiology: Optimized Protocol for Culture of Mouse Fetal Hindbrain Neurons

**DOI:** 10.3390/cells14110758

**Published:** 2025-05-22

**Authors:** Hadrien Glibert, Laure Bridoux, Maëlle Palate, Coralie Piget, Marie-Thérèse Ahn, Roberta Gualdani, Ana Domínguez-Bajo, Frédéric Clotman, Filippo M. Rijli, Françoise Gofflot

**Affiliations:** 1 Louvain Institute of Biomolecular Science and Technology, UCLouvain, B-1348 Louvain-la-Neuve, Belgium; hadrien.glibert@uclouvain.be (H.G.); ana.dominguez-bajo@inserm.fr (A.D.-B.); frederic.clotman@uclouvain.be (F.C.); 2 Laboratory of Cell Physiology, Institute of Neuroscience, UCLouvain, B-1200 Brussels, Belgium; roberta.gualdani@uclouvain.be; 3 Turing Center for Living Systems, Aix Marseille University, INSERM, INMED U1249, 13723 Marseille, France; 4 Friedrich Miescher Institute for Biomedical Research, Fabrikstrasse 24, 4058 Basel, Switzerland; filippo.rijli@fmi.ch; 5 University of Basel, 4003 Basel, Switzerland

**Keywords:** hindbrain, neuronal culture, glial cell, astrocyte, hydroxytamoxifen, synapse

## Abstract

Primary cultures of neural cells are important key tools for basic and translational neuroscience research. These primary cell cultures are classically generated from the rodent brain hippocampus or cortex and optimized for enrichment in neurons at the expense of glial cells. Importantly, considerable differences exist in neuronal cell populations and in glial cell contribution between different brain regions. Because many basic and translational research projects aim to identify mechanisms underlying brainstem neuronal networks that affect major vital functions, primary cultures representative of cell populations present in the hindbrain are required. However, the preparation of primary cultures of brainstem/hindbrain neurons is scarcely described in the literature, limiting the possibilities for studying the development and physiology of these brain regions in vitro. The present report describes a reliable protocol to dissociate and culture in vitro embryonic mouse fetal hindbrain neurons in a defined culture medium, while control of astrocytes’ expansion was attained by using a chemically defined, serum-free supplement, namely CultureOne™. The neuronal cells maintained according to this protocol differentiate and, by 10 days in vitro, they develop extensive axonal and dendritic branching. Using immunofluorescence, we further characterized the different cell populations and neuronal subtypes. Patch–clamp recordings demonstrate the excitable nature of these neurons, while colocalization of pre- and postsynaptic neuronal markers showed that neurons form mature synapses, suggesting the establishment of functional networks in vitro. The cultures produced by this method show excellent reproducibility and can be used for molecular, biochemical, and physiological analyses, as illustrated here for tamoxifen-induced Cre recombination in genetically-modified neural cells.

## 1. Introduction

The brainstem, composed of the midbrain, pons, and medulla oblongata, sustains fundamental homeostatic functions, such as the control of breathing, heart rate and blood pressure, control of consciousness, and sleep [1,2,3,4,5]. It is also of prime importance in the conveyance of motor and sensory pathways from the rest of the brain to the body, and from the body back to the brain [6]. In view of its essential roles in major vital functions, models allowing researchers to investigate brainstem/hindbrain mechanisms in health and disease are required [1]. However, until recently, the study of the hindbrain has been neglected, mainly due to the difficulty in approaching it experimentally: it lies in a relatively inaccessible region of the nervous system and its structure is devoid of useful landmarks, consisting mainly of an apparently diffuse, reticular network. Over the last 15 years, our understanding of hindbrain circuitry has advanced significantly, thanks to transgenic mice generation, electrical and optical recording techniques and, more recently, computational methods [7,8,9,10,11,12], making it possible to explore previously inaccessible questions. However, reliable procedures for primary cultures of neural cells, which serve as important tools in biomedical research, are still missing for the murine hindbrain.

While neuronal cell lines have been key in characterizing neuronal cell physiology, primary cultures offer a relevant alternative to tumor-derived and immortalized cell lines as they are more likely to recapitulate the properties of neuronal cells in vivo [13]. As such, primary neuron culture from the embryonic rodent hippocampus or cortex has been one of the most fundamental methodologies for modern neurobiology. In contrast, almost no protocols are available for the brainstem. As primary cultures recapitulate the regional specificity and diversity present in the brain, the absence of satisfactory protocols for obtaining cultures from the hindbrain is a limitation to in vitro analyses of neuron–neuron interactions, synapse formation and functioning, as well as neuron–glial cell relationships in this specific region. Indeed, the hindbrain is notable for its diverse neuronal cell types and wide range of neurotransmitters. In addition to acetylcholine, glutamate, and gamma-aminobutyric acid (GABA), hindbrain neurons produce other neuromodulators, like glycine and monoamine neurotransmitters [5,14,15,16]. Both neuronal subtypes and glial cells vary between different brain regions. Astrocytes, in particular, exhibit regional heterogeneity in cellular, molecular, and physiological aspects, indicating significant molecular and functional differences [17,18]. Oligodendrocytes and microglia are also expected to vary from one brain region to another, if only in terms of density [19].

Our objective is, thus, to develop a protocol that circumvents the technical constraints of primary culture preparation from the hindbrain while preserving neuronal and glial populations specific to this region, yet while avoiding the expansion of glial populations. In 2001, a protocol for preparing primary brainstem neurons was described for rats, but so far reliable protocols are missing for the culture of primary brainstem neurons from mice [20,21,22]. Building upon the rat protocol, we report here an optimized protocol that facilitates the reliable isolation and culture of fetal hindbrain neural cells from mice, which we believe to be suitable for research on hindbrain-specific neural populations.

## 2. Materials and Methods

### 2.1. Animals

Mice were maintained in a conventional facility and fed in standard conditions (mice maintenance and mice breeding diets, Carfil Quality, Turnhout, Belgium), on a 14 h light/10 h dark cycle. Food and water were available ad libitum. Experimental procedures on animals were approved by the animal ethics committee of the UCLouvain under number #122803 and performed in accordance with the European directive 2010/63/UE. Fetal brainstems were dissected from fetuses obtained from time-mated mice (incipient congenic background, 97% C57Bl6/J). Mice were mated overnight, and successful mating was confirmed by the presence of vaginal plugs the next morning; this was defined as embryonic day (E)0.5. Pregnant mice were euthanized by cervical dislocation, while fetuses were decapitated before dissection.

The transgenic CMV-CreER^T2^ have been described elsewhere [23,24]. Ai14 Rosa26R-tdTomato reporter mice have been described elsewhere [25]. Genotyping of animals was accomplished by polymerase chain reaction (PCR) using a Taq Master mix (M7423, Promega, Madison, WI, USA) with the primers and PCR program described in Table 1.

### 2.2. Preparation of Cell Culture Media

Several solutions and media were required for the dissection and culture procedures. They were prepared prior to the dissection, sterile-filtered through 0.22 µm filters (SLGP033RB, Sigma, Burlington, VT, USA), and kept at 4 °C. Media were warmed to 37 °C in a water bath before use. Unless otherwise specified, components were supplied by Thermo Fisher Scientific (Waltham, MA, USA). Solution 1 was composed of Hank’s balanced salt solution (HBSS) without Ca^2+^/Mg^2+^ (14170-088). Solution 2 was composed of 50 mL HBSS with Ca^2+^/Mg^2+^ (14025-050), 500 µL HEPES 1M (15630056) and 500 µL sodium pyruvate 100 mM (11360039). For neuron culture, the NB27 complete medium was prepared by mixing 50 mL Neurobasal™ Plus Medium (A3582901) with 1 mL B-27™ Plus Supplement (A3582801), 62.5 µL L-glutamine 200 mM (25030-024), 62.5 µL GlutaMax 200 mM (35050061), and 100 µL penicillin–streptomycin 5000 U/mL (15070063). CultureOne™ supplement 100× (A3320201) was used as an additive to the complete medium and incorporated at the third day in vitro at 1× concentration. Fetal bovine serum (FBS) (F7524) was decomplemented at 56 °C for 30 min before use.

### 2.3. Fetal Tissue Dissociation

All steps are performed with sterile instruments and consumables. Primary hindbrain cultures were established from E17.5 fetuses. Pregnant mice were euthanized as described above. E17.5 fetuses were removed from the uterus, maintained at 37 °C, and euthanized by decapitation according to guidelines. Brains were collected and put in petri dishes containing sterile phosphate-buffered saline (PBS-18912014) for further dissection. Brainstems were isolated from the whole brain under a dissecting microscope; the cortex, remnants of the cervical spinal cord, and cerebellum were first removed to isolate the brainstem. Finally, the hindbrain was separated from the midbrain by cutting from the dorsal fold separating the two regions towards the ventral pontine flexure (Supplemental Figure S1). In the first experiments, cortices were also collected as control for hindbrain cultures. Lastly, remaining blood vessels and meninges were carefully removed. Dissected samples were then transferred to 15 mL tubes containing 4 mL of Solution 1 and pooled up to 4 hindbrains per tube. All subsequent experiments were carried out under sterile conditions.

Each 15 mL tube was treated as follows. Hindbrains were first mechanically dissociated with a plastic sterile transfer pipette (612-1683, Avantor, PA, USA) into 2–3 mm^3^ pieces. The tissue matrix was loosened by adding 350 µL of Trypsin 0.5% and EDTA 0.2% (T4174, Sigma, Burlington, VT, USA) per tube. Tubes were gently mixed and incubated for 15 min at 37 °C. Loosened tissues were then mechanically dissociated in 10 up-and-down motions using a long-stem glass Pasteur pipette (612-1702, VWR). Tubes were incubated again for 5 min at 37 °C, then triturated 10 times with a fire-refined long-stem glass Pasteur pipette (allowing the diameter to be reduced from 750 µm to 675 µm). Then, 4 mL of Solution 2 was added to each tube, which was gently mixed by inverting 2–3 times, then left to settle 2–3 min to allow large cell debris to settle to the bottom. The cell suspension was then carefully collected, leaving out the debris, and transferred to a 15 mL Falcon tube containing 7 mL of warm decomplemented FBS. Cells were pelleted by centrifugation at 300g for 10 min and resuspended in the appropriate amount of NB27 medium. Resuspending the pellets in 2 mL of medium per hindbrain yielded 1.34·10^5^ ± 0.38·10^5^ cells/mL, as counted for five independent cultures.

### 2.4. Fetal Cell Culture

Depending on the objectives of the culture and subsequent procedures, dissociated cells were plated onto plastic multi-well plates or on glass coverslips previously coated with poly-L-lysine (PLL; P1399, Sigma, Burlington, VT, USA). Coating was performed beforehand by incubating circular coverslips (631-1578, Avantor, PA, USA) with 500 µL of 10 µg/mL of PLL diluted in 1XPBS at 37 °C for a minimum of 30 min. The excess coating solution was removed, and the coverslips were then rinsed 2 times with PBS. Cells were seeded at a density of 100,000–120,000 per well in 24-well culture plates (approximately 50,000–65,000 cells/cm^2^) in 1 mL of complete growth medium. Cells were incubated in a controlled atmosphere of 95% air, 5% CO_2_, and 97% humidity at 37 °C. After 3 days in vitro (DIV), the medium was replaced with the complete growth medium supplemented with CultureOne^TM^ (Thermo Fisher Scientific, Waltham, MA, USA) to avoid glial cell overgrowth. Cells were then cultured to 10 DIV and either harvested or fixed depending on the subsequent procedures.

### 2.5. Immunofluorescence

For immunofluorescence, coverslips were fixed with 4% paraformaldehyde (PFA) in 1XPBS. After rinsing wells with PBS, 500 µL of the PFA solution was added to each well and incubated for 20 min at room temperature (RT). Coverslips were then rinsed twice with PBS and left immersed in PBS for conservation at 4 °C.

Coverslips were blocked in a solution of 5% milk (Regilait 0% skim milk, Saint-Martin-Belle-Roche, France) in TBS-Tx (Tris-buffered saline 0.05 M–Triton X-100 (T8787, Sigma, Burlington, VT, USA) 0.1%, pH 7.4) for 1h at RT. Cells were incubated overnight at 4 °C with primary antibody diluted in TBS-Tx 1% skim milk in a humid chamber (for antibody dilution, see Table 2). After incubation with primary antibody, cells were rinsed 3 times for 5 min in TBS-Tx and incubated 1h at RT with the appropriate secondary antibodies (1:500 in TBS-Tx) (Table 3). After 2 washes of 5 min in TBS-Tx, coverslips were stained with DAPI in TB (Tris-buffered saline 1M, DAPI 25 µg/mL, pH 7.5) then mounted with Fluorescence Mounting Medium (S302380-2, Dako, Agilent, SC, USA). Samples were then imaged with either a Zeiss AxioSkop2 fluorescence microscope or a Zeiss AXIO Observer Z1 (Zeiss, Oberkochen, Germany).

### 2.6. Electrophysiological Analyses

Patch–clamp recordings of hindbrain-derived neurons were carried out as previously described [26,27]. Voltage clamp experiments were performed using an EPC-9 amplifier controlled by Patchmaster v2x90.3 software (HEKA Elektronik, Lambrecht, Germany). An AgCl wire was used as a reference electrode. Solutions were applied to the cells via a homemade gravity-fed perfusion system, connected by a 5-way manifold, to a RC25 perfusion chamber (Warner Instruments, Hamden, CT, USA). The patch pipettes were pulled with a resistance of 3–5 MΩ using a DMZ-Universal Puller (Zeitz Instruments, Munich, Germany). The extracellular solution had the following composition (in mM): 140 NaCl, 5 KCl, 2 CaCl_2_, 1 MgCl_2_, 10 glucose, and 10 HEPES, pH 7.4. The pipette solution had the following composition (in mM): 135 KCl, 1 K_2_ATP, 1 MgATP, 2 EGTA, 1.1 CaCl_2_, 5 glucose, and 10 HEPES (pH 7.2). Seven neurons were tested in one culture at 10 DIV, of which five responded.

### 2.7. RT-qPCR

RNA from primary cultures and dissociated murine hindbrains was extracted using a Roche High Pure RNA Isolation Kit (11828665001, Roche, Bâle, Switzerland) according to the manufacturer’s instructions. Reverse transcription was performed using a reverse transcription kit (1708891, BioRad, Hercules, CA, USA) according to the manufacturer’s instructions. Gene expression was assessed by qPCR on a StepOne+ apparatus (Applied Biosystems, Waltham, MA, USA) using SYBR Green (204143, QIAGEN, Hilden, Germany) as the detection method, and the geometric mean of two reference genes was used as internal normalization reference. For in vitro samples, the stability of 6 candidate genes (*H2a*, *36b4*, *Hprt*, *Rpl32*, *Tbp* and *Ppia*) was assessed using the RefFinder web tool [28] (https://www.ciidirsinaloa.com.mx/RefFinder-master/, (accessed on 14 April 2025)) resulting in the selection of *H2a* and *Ppia* as the most suitable genes. To allow the comparison of in vitro and in vivo samples, *36b4* [29] was tested along *H2a* and *Ppia* in a stability test to identify the most stable reference genes pair, leading to the selection of *H2a* and *Ppia*. Data were analyzed and presented using the ΔΔC_t_ method. The primer pairs used are presented in Table 4. Each primer pair was tested for an amplification efficiency between 90 and 110%.

### 2.8. Cre-Mediated Recombination in Culture

Primary cultures were treated with a final concentration of 1 µM of 4-Hydroxy-Tamoxifen (4-OHT; H7904, Sigma, Burlington, VT, USA). Prior to use, a stock solution of 10 mM 4-OHT in absolute ethanol (EtOH) was heated for 10min at 65 °C. Then, 100 µM working solutions were prepared by dilution in EtOH. Wells were subsequently treated with 10 µL of working solution or 10 µL of EtOH alone for controls. The results show the cultures treated according to this procedure at both 1 and 2 DIV.

### 2.9. Experimental Design and Statistical Analyses

Statistical analyses were performed on 4 independent cultures. Regarding RT-qPCR data, the means of ΔC_t_ were compared using a paired *t*-test. Cellular quantifications were analyzed using a mixed binomial generalized linear model, with the CultureOne™ treatment as fixed effect and the culture as random effect.

R [30] and Rstudio IDE [31] were used for statistical analyses and graphical representation. ggplot2 [32] (v3.5.1) was used for graphical representation, and lme4 [33] (v1.1-37) for mixed generalized linear modeling.

## 3. Results

### 3.1. Isolation of Cells from the Fetal Mouse Hindbrain

The culture of neuronal cells is particularly challenging because mature neurons no longer undergo cell division, which is why many primary cultures are established from fetal brains. As a preliminary step, we compared primary cultures from hindbrains dissected either at fetal or postnatal day 1 and observed a significantly lower abundance of all cell types, in particular neurons, in cultures from postnatal hindbrains). All the cultures described here were, therefore, produced from E17.5 fetal hindbrains.

**Figure 1:**
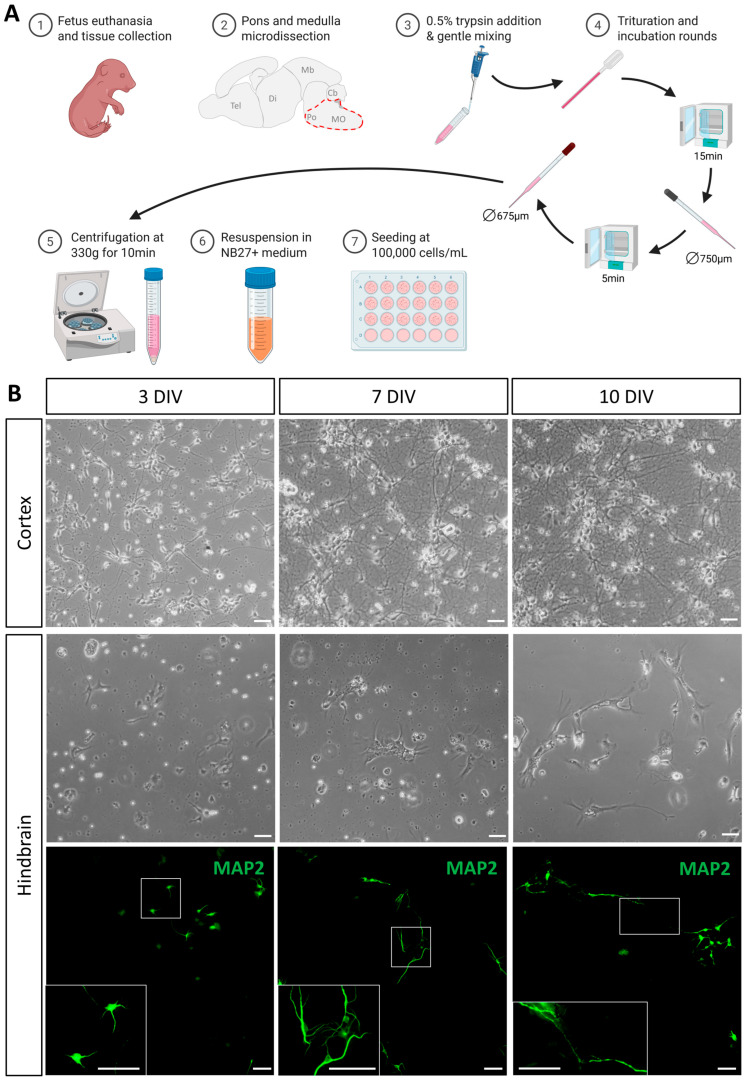
Graphical protocol and cell expansion in culture. (**A**) Graphical representation of the microdissection, dissociation, and culture protocol. (**B**) Expansion of cells at different timepoints during culture: 3 days in vitro (DIV), 7 DIV, and 10 DIV. First and second rows represent phase contrast images of cortex and hindbrain-derived cultures, respectively. Images in the third row represent immunofluorescence of hindbrain-derived cultures stained for MAP2. The frame in the main image identifies the enlarged region in the inset at the bottom left of each picture. Scale bar is 50 µm.

The overall procedure is outlined in Figure 1A. For comparison and procedural quality control, cortical cells from the same brains were prepared and cultured in parallel, but trituration of cortical and hindbrain tissue was performed separately. Although the cell suspension was carefully collected at the end of the dissociation process, the presence of cell debris prevented the use of automated counting devices. Using manual counting with the Bürker method, the protocol generally yields a density of around 130,000 cells/mL when resuspending in 2 mL per hindbrain collected (see Section 2). Cell counts using the Kova method gave similar results. By way of comparison, resuspension of two cortical hemispheres yields around 800,000 cells/mL. To minimize inter-individual and/or inter-litter variability, cell suspensions obtained from all dissected hindbrains were pooled together before seeding, ensuring homogenous populations in the different culture wells of a given experiment.

Cells were visualized by phase contrast optics and immunofluorescence using anti-microtubule-associated protein 2 (MAP2) antibody at different time points (Figure 1B). As expected, our procedure resulted in the successful culture of cortical-derived cells which quickly became adherent and derived neurites with 2–3 DIV. At 7 DIV, many of these cells developed long projections and established long-distance connections, resulting in a dense network of neurites at 10 DIV. In comparison with cortical-derived cells, and in line with challenges described above, less cells dissociated from the hindbrain were observed at the onset of cultures. Nevertheless, these hindbrain-derived cells attached to the coverslip within one day in culture and most of them began to produce neurites within the first few days, with short extensions being clearly visible at 3 DIV. At 7 DIV, most of these cells showed a generalized neural morphology with rounded cell bodies and well-developed neurites, forming small clusters and establishing local connections. At this stage, neuronal morphology was better visualized in immunofluorescence, as neurons extend preferentially in close vicinity with astrocytes. With only one change of medium at 3 DIV, the optimal culture time to obtain thriving neuronal populations appeared to be 10 days (Figure 1B). At that stage, more cells with longer processes and neuronal morphology were observed, establishing long-distance connections. Beyond this timing, neuronal morphology appeared impacted, probably due to medium depletion. In conclusion, the procedure consistently yielded viable cell cultures, including well-developed neurons displaying features characteristic of their in vivo cell morphology within 10 days of culture.

### 3.2. Characterization of the Hindbrain Neuronal Populations Obtained In Vitro

**Figure 2:**
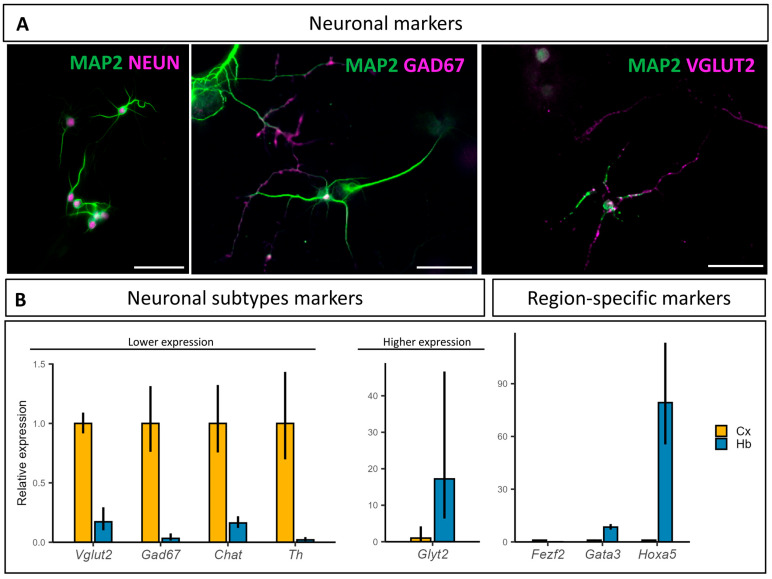
Neuronal populations characterization at 10 DIV: (**A**) Neurons are postmitotic and mainly glutamatergic and GABAergic. Antibodies targeting MAP2 are in green, and those targeting NEUN, GAD67, and VGLUT2 are in magenta. Scale bar is 50 µm; (**B**) mRNA relative expression of neuronal subtypes and brain region-specific markers in cortical (Cx) and hindbrain (Hb) primary cultures at 10 DIV. *Vglut2*: Vesicular glutamate transporter 2, *Gad67*: Glutamate decarboxylasse 67, *Chat*: Choline acetyltransferase, *Th*: Tyrosine hydroxylase, *Glyt2*: Glycine transporter 2, *Fezf2*: FEZ family zinc finger 2, *Gata3*: GATA binding protein 3, and *Hoxa5*: Homeobox A5. (N = 4).

First, to follow the neurons’ development in culture, the pan-neuronal marker, neuronal nuclei (NEUN), was used to label postmitotic neurons nuclei. To analyze the neuronal morphology, the anti-microtubule-associated protein 2 (MAP2) antibody was used to stain neuronal bodies and dendrites. As observed at 10 DIV, neurons in culture are marked by NEUN and showed well-developed extensions labelled with MAP2 (Figure 2A), indicating a mature postmitotic state. Due to the large variety of neuronal cell types and a large number of neurotransmitters used in the brainstem, different markers were used to identify the subpopulations present in the primary culture. The vast majority of neurons present in our cultures were excitatory glutamatergic or inhibitory GABAergic neurons. Indeed, the VGLUT2 and GAD67 mouse monoclonal antibodies displayed their distinctive punctate staining in numerous neuronal projections (Figure 2A). Other antibodies were used to identify other neuronal subsets present in monoaminergic nuclei, such as the choline acetyltransferase (CHAT) antibody for cholinergic neurons, the tyrosine hydroxylase (TH) antibody for catecholaminergic (dopaminergic and noradrenergic) neuron populations, and an anti-serotonin antibody for serotoninergic neurons. However, we were unable to detect these modulatory neurons in our cultures using immunofluorescence, which might be explained by their presence in very small numbers (small masses of thousands of neurons).

mRNA quantification of neurotransmitter-specific markers showed a lower relative expression in the hindbrain compared to the cortical primary cultures for *Vglut2*, *Gad67*, *Chat*, and *Th*, and much higher expression for *Glyt2* (Figure 2B). Noteworthily, *Glyt2* expression was below the RT-qPCR detection threshold in the cortical culture samples for two of the four replicates. We also investigated the relative abundance of the hindbrain-specific transcription factors *Hoxa5* and *Gata3*, as well as the telencephalic marker, *Fezf2*, all of which showed an expected region-specific relative abundance (Figure 2B). Similar to *Glyt2*, *Hoxa5* expression was below the RT-qPCR detection threshold in the cortical cultures for three of the four replicates.

Overall, these data suggest that neurons present in culture after 10 DIV are able to form connected networks and that they retain their hindbrain-derived identity.

### 3.3. Synaptic Properties of Hindbrain-Derived Primary Neurons

**Figure 3:**
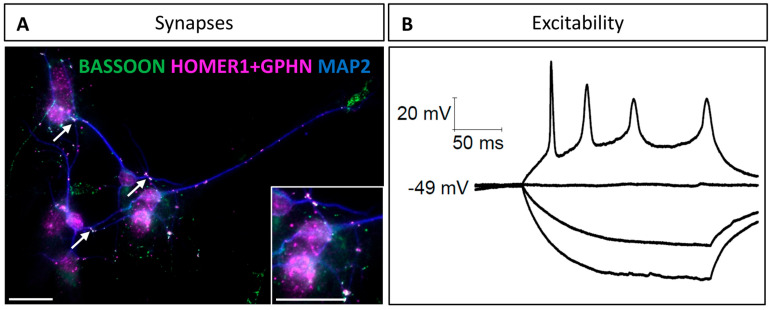
Neurons in cultures form synapses and elicit action potential by 10 DIV: (**A**) Colocalization between presynaptic (BASSOON) and postsynaptic (HOMER1 and GEPHYRIN) proteins. BASSOON is in green, and both GEPHYRIN and HOMER1 are labeled in magenta. MAP2 is in blue. Synapses are indicated by magenta and green labelling colocalization (white arrows). Scale bar is 25 µm. (**B**) Representative traces from a hindbrain-derived neuron in response to depolarizing current steps of 10 pA, showing a current threshold (i.e., the minimum amplitude of a current step required to evoke an action potential) of 30 pA (N = 1).

Next, we investigated the synaptic features of neurons based on the colocalization of pre- and postsynaptic neuronal markers. This method was described and used by Verstraelen et al. to identify mature synapses [34]. We selected BASSOON as a pan-presynaptic protein, as this protein is mainly found in active zones of both excitatory and inhibitory synapses [35]. For the postsynaptic markers, two different proteins were selected to discriminate between excitatory and inhibitory synapses. HOMER1 is specific to excitatory synapses and contributes to their structure by binding metabotropic glutamate receptors [36]. A scaffold protein known as GEPHYRIN was selected as the inhibitory counterpart of HOMER1. Originally discovered as a scaffold protein in glycinergic inhibitory synapses [37], GEPHYRIN is now recognized as the core structural protein of inhibitory synapses in general [38]. Via co-immunodetection of BASSOON and either HOMER1 or GEPHYRIN, we were, therefore, able to identify the presence of mature glutamatergic and GABAergic synapses, respectively. At 10 DIV, many spots of colocalizations between presynaptic and postsynaptic markers were observed (Figure 3A), confirming that connections between neurons in culture result in mature synapse establishment.

Finally, to check whether these hindbrain-derived neurons were functional, we examined their ability to elicit an action potential. Using patch–clamp recording in the current clamp configuration with 10 pA depolarizing current steps, we were able to demonstrate the excitable nature of these neurons (Figure 3B). These results indicate that the neurons dissociated from fetal hindbrains and maintained in culture using our protocol are able to form mature synapses and elicit action potentials at 10 DIV, and are, therefore, fully functional for studying neuronal differentiation and synaptogenesis.

### 3.4. Glial Populations

**Figure 4:**
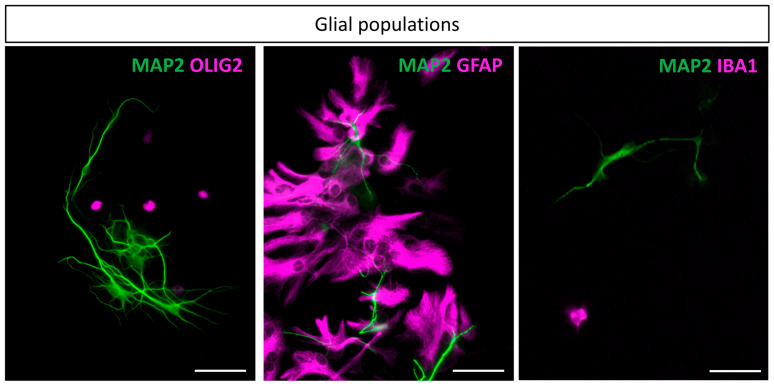
Glial populations at 10 DIV: The three main glial cell types found in the culture. Neuronal bodies and dendrites are marked on every image using MAP2 antibody. From left to right, in magenta, are antibodies targeting Olig2, GFAP, and IBA1. Scale bar is 50 µm.

Next, different molecular markers were used to identify glial cell population in cultures from fetal hindbrains at 10 DIV. The anti-OLIG2 antibody was used to specifically stain the oligodendrocyte lineage, and the anti-glial fibrillary acidic protein (GFAP) was used as an astrocyte marker. Finally, IBA1, an EF-hand domain protein, was characterized to be expressed in the monocytic lineage and its expression in the brain was characterized to be restricted to microglia [39]. Our cell dissociation procedure successfully produced representative glial cells in culture, namely astrocytes, oligodendrocytes, and microglia (Figure 4). Of note, astrocytes were particularly enriched in the cultures, as detailed in the section below. While they are known to be highly heterogeneous throughout the brain, our culture led to a vast majority of protoplasmic astrocytes and a minority of fibrous astrocytes. In contrast, in the cortical cultures, fibrous astrocytes were the majority. Oligodendrocytes and microglia were also present in the cultures, although at a lower abundance than astrocytes.

### 3.5. Impact of CultureOne™ Supplement

Originally developed to favor differentiated cells maintenance in neural stem cells culture, the CultureOne™ supplement appeared effective in inhibiting glial cells proliferation, while still allowing their survival and, most importantly, leaving the neurons unaffected [40]. To assess the benefits in hindbrain-derived cultures, we compared cultures with and without supplement added in the medium at 3 DIV.

**Figure 5:**
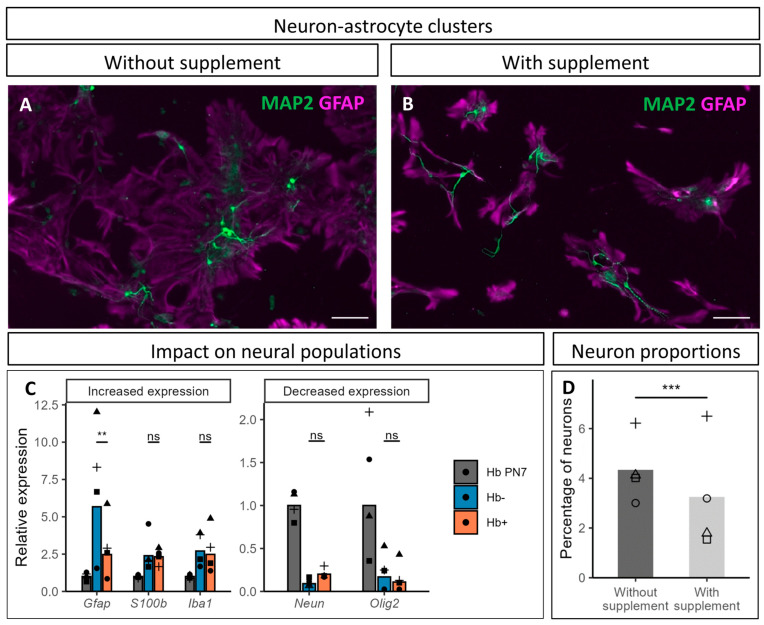
Impact of CultureOne™ supplement on astrocytic and neuronal populations at 10 DIV. (**A**,**B**) MAP2 (green) and GFAP (magenta) immunostaining on cultures without and with supplement, respectively. Scale bar is 100 µm. (**C**) RT-qPCR analysis of neural cell markers at 10 DIV. Hb PN7: pool of two isolated hindbrains at postnatal day (PN) 7, Hb-: cell culture without the supplement, Hb+: cell culture with the supplement. Cultures and hindbrain collection were repeated four independent times (N = 4) and values of each culture are represented by different symbols: cross, square, triangle, and circle. *Gfap*: glial fibrillary acidic protein, *S100β*: S100 calcium binding protein, *Iba1*: allograft inflammatory factor 1, *Neun*: neuronal nuclei, *Olig2*: oligodendrocyte transcription factor 2. Statistical comparisons were made between Hb- and Hb+ samples using a paired *t*-test. (**D**) Neuronal percentage of four different cultures treated or not with the supplement (values of each culture are represented by different symbols: cross, square, triangle, and circle). Statistical analysis was performed using a mixed binomial generalized linear model. “ns”: non-significant difference, **: *p*-value below 0.01, ***: *p*-value below 0.001.

The supplement appeared to strongly impact astrocytic development and morphology (Figure 5A,B). In supplemented cultures, astrocytes showed less expansion and reduced development of their cellular processes. Their tendency to spread out and form plaques also seemed to decrease. These morphological changes had no impact on the local association between neurons and astrocytes, and did not appear to alter neurite development.

To further evaluate the impact of the supplement on cell populations present in the culture, we examined the relative expression of neural cells markers, using *Neun* for the neurons, *Olig2* for the oligodendrocytes, *Iba1* for the microglia, and two markers of different astrocytic populations, namely *Gfap* and *S100*β (Figure 5C). Their expression was compared between cultures with and without addition of CultureOne™ supplement (Hb+ and Hb-, respectively), while the expression was also measured directly in cells obtained from hindbrains dissected from PN7 mouse brains (Hb PN7). When compared to Hb PN7 samples, clear tendencies were highlighted in cultures, regardless of the treatment with the supplement. The expression of both astrocytic markers was higher in cultures, as was *Iba1* expression, while *Olig2* expression was lower. Unsurprisingly due to the postmitotic state of the neurons in culture, *Neun* expression also decreased, although a tendency suggesting a positive impact of the supplement on neuronal population was highlighted.

Regarding the impact of the supplement on the expression of these markers, only *Gfap* expression was significantly impacted by the treatment, which could be related to the reduction in the cellular extension/surface of astrocytes observed in immunofluorescence (Figure 5A,B), and/or to a reduced number of astrocytes due to reduced proliferation. The expression of the other markers tested did not show significant difference in the presence of the supplement, although a slight increasing trend in *Neun* expression was observed. Conversely, when assessing the proportion of neurons directly, their proportions appeared to be negatively impacted by the supplement (Figure 5D). This result was, however, observed only in two of the four cultures counted, while the neuronal proportion remained unaffected in the other two.

While the supplement seems to mitigate astroglial extension/growth, its impact on neurons remains unclear. Qualitative evaluation of the cultures suggests a beneficial impact for the cultures, with limited expansion of astrocytes and a slightly positive effect on neurons, but attempts to quantify this observation led to opposite results for neurons (no effect or a reduction). These data will be discussed in more detail below.

### 3.6. Culture of Hindbrain Neural Cells from Transgenic Mice and In Vitro Induction of Cre Recombination

**Figure 6:**
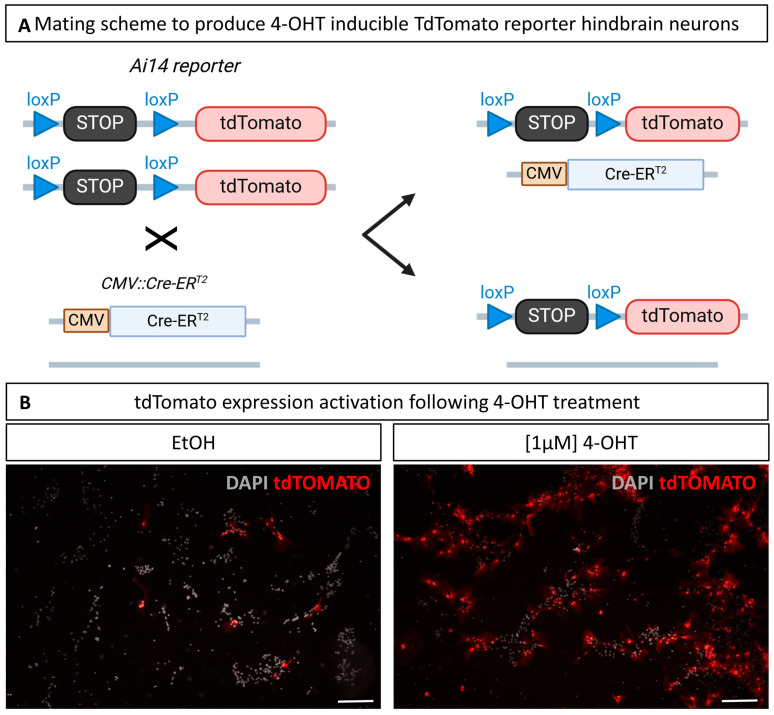
4-Hydroxy-tamoxifen (4-OHT) treatment induces Cre recombination in primary cultures derived from Ai14;CMV-CreER^T2^ mice. (**A**) Hindbrains are pooled from litters obtained by mating homozygous Ai14 males and heterozygous CMV-CreER^T2^ mice; (**B**) primary cultures treated with EtOH only, as a control of Cre background activity, or with 1 µM 4-OHT at 1 and 2 DIV. Red signal is the tdTomato endogenous signal imaged at 10 DIV. Scale bar is 200 µm. N = 3.

Having established that our hindbrain-cultured neurons develop networks with mature synapses and are able to generate action potential, we wished to illustrate the relevance of these cultures to answer scientific questions. As mentioned, neuronal cultures provide a simplified model for better understanding neuronal differentiation and function, enabling molecular and pharmacological manipulations. The ability to develop and manipulate cells specifically derived from the hindbrains of transgenic mouse lines is therefore a major asset for understanding the molecular mechanisms underlying hindbrain processes. Here, we tested their ability to efficiently and timely induce gene inactivation in culture using the tamoxifen-inducible Lox/CreER^T2^ system. Fetuses were obtained by breeding homozygous Ai14 males with females heterozygous for the constitutively expressed inducible recombinase CreER^T2^ (Figure 6A). Cultures were derived from a pool of the whole litter, whose fetuses all harbored the *tdTomato* reporter transgene, but only half carried the *CreER^T2^* transgene.

To achieve recombination, primary cells were treated twice with 1 µM 4-hydroxy-tamoxifen (4-OHT) dissolved in ethanol (EtOH) at both 1 DIV and 2 DIV. As a control, wells were treated with the same volume of EtOH without 4-OHT to visualize the background Cre activity, sometimes referred to as leaking, and to account for potential EtOH-specific toxicity. As illustrated in Figure 6B, 4-OHT treatment induced *tdTomato* expression in a large proportion of the cells, suggesting high CreER^T2^-induced recombination efficiency. A tdTomato signal was also detected in a few scattered cells in the EtOH-treated wells, likely due to leaking of the CreER^T2^, likely resulting from the highly active CMV promoter. Qualitative assessment of those cultures showed no visible impact of the 4-OHT treatment on the neuronal morphology or culture development overall. This experiment demonstrated the value of these cultures for testing, in an in vitro model, hypotheses derived from in vivo transgenic models on the functions of some genes in the processes of neuronal differentiation, neurite growth, or synaptogenesis, making it possible to control the temporal dynamics of inactivation and facilitate analysis in a 2D system.

## 4. Discussion

### 4.1. Neuronal Populations

Using the optimized protocol described in this article, we successfully obtained in vitro mature and functional hindbrain neurons associated with glial cell populations specific to this region. These neurons are differentiated, establish neuronal networks, form mature synapses, are able to emit action potential, and predominantly use GABA or glutamate as their neurotransmitters.

Despite the presence of serotonergic (i.e., raphe nuclei), catecholaminergic (locus coeruleus), and cholinergic neurons in the hindbrain, neither serotonin, tyrosine hydroxylase nor choline acetyltransferase could be detected in the cultures using immunofluorescence. Although these neuromodulatory neurons project their axons into large areas of the central nervous system in vivo and, thus, influence numerous behaviors, they are present in very low numbers, which could explain their scarcity in our cultures. As an example, the number of neurons in the locus coeruleus of the mouse brain is estimated between 1300 and 1500 [41,42], which represents less than 1% of cells dissociated and cultured from a hindbrain. With regard to noradrenergic neurons, we cannot exclude that part of the locus coeruleus has been omitted from the dissection, as the cutting axis passes close to the third rhombomere where the locus coeruleus is located. If required, enrichment of specific neuronal populations in these cultures could be achieved through a negative selection process using magnetic tagging of the major neuronal populations (glutamatergic or GABAergic) followed by sorting onto a ferromagnetic sphere column [22], as well as by targeting specific nuclei via brain slices microdissection [43]. On the other hand, glycinergic neurons are found in many nuclei of the brainstem and, although their expression remained low, *Glyt2* mRNA expression was enriched by 15-fold in the hindbrain cultures compared to the cortical cultures. As the hindbrain cultures contain a higher proportion of glial cells than cortical cultures, we expect the mRNA quantification of neurotransmitter-related markers to exhibit a downward bias in the hindbrain samples. Taking this into account would increase the difference in expression of *Glyt2* mRNA between the hindbrain and cortical samples, while reducing the gap in the other markers.

### 4.2. Glial Cells Populations

The procedure described here produced representative glial cells in culture, namely astrocytes, oligodendrocytes, and microglia. Astrocytes are the most abundant, with a predominantly protoplasmic morphology, while oligodendrocytes and microglia are found at a lower abundance, which appears to be below that observed in vivo, particularly for oligodendrocytes.

Long considered as contaminating cells that needed to be excluded for the purity of neuronal culture, we argue that the glial cells participate in a more physiologically relevant environment than pure neuronal cultures. These cells are more than relevant for a lot of research applications, as it is now widely accepted that astrocytes are important players in the tripartite synapse and synaptogenesis processes [44]. They regulate synapse formation through various mechanisms, one of which is the secretion of extracellular factors. As first demonstrated by the Barres team, macroglial cells of the retina drastically increase the number and amplitude of spontaneous excitatory currents in cultured retinal ganglion cells (RGCs). Additionally, RGCs cultured in presence of glial cells formed, on average, 2.3 times more synapses than RGCs alone [45]. These results are supported by another study reporting a similar increase in synapse number by treating primary neurons from various brain regions with an astrocyte-conditioned medium [46]. In addition to synapse formation, other astrocyte-secreted factors called glypicans 4 and 6 regulate synapse maturation [47]. More recently, microglial cells have also been highlighted for their endogenous contribution to neuronal activity and synapse regulation [48]. Taking these findings into account, it appears critical to conserve glial cells in the cultures, especially for synapse-related research.

Regarding the oligodendrocytes, our results showed that the population was quite underrepresented in cultures compared to their in vivo proportions. While their number were not quantitatively assessed, Olig2-positive cell nuclei were few in the cultures, contrasting with the oligodendrocyte–astrocyte balance recently estimated for the in vivo murine hindbrain [19,49,50]. This observation could result from the dissociation protocol, which would have a differential impact on cell types, and variations in their proportions are expected. The dissociation process was indeed adapted to the well-developed myelination of the hindbrain, which was confirmed by the amount of debris generated in this process for the hindbrain compared to the cortex. The smaller number of oligodendrocytes does not seem to have any impact on the establishment and maturation of neuronal networks in culture.

Nevertheless, in culture, astrocytes and glia in general lack the growth regulation found in vivo and tend to overgrow the neurons. To overcome this issue, antimitotics are frequently used in the culture medium [21,51,52,53]. However, reports indicate neurotoxicity following the use of these agents [54,55,56]. We chose the commercial alternative of the CultureOne^TM^ supplement, originally developed for improving neural stem cells differentiation [40]. This supplement improved our cultures by reducing the formation and amplitude of protoplasmic astrocytes plaques (Figure 5A,B), without impacting neurite extension and neuronal connections, which may provide advantages for tracking and analyzing fine structures, such as synapses. As GFAP expression was shown to be modulated by redox conditions [57], while the difference in astrocyte morphology and *Gfap* mRNA expression could be explained by antioxidative properties of the supplement. Should this be the case, the addition of the CultureOne™ supplement would confer protective benefits on astrocytes, and consequently, on neurons. While this supplement is expected to have minimal impact on redox balance (Thermo Fisher Scientific communication), the exact composition of the CultureOne™ supplement is unknown, and this hypothesis remains speculative. Regarding the effects of the supplement on neurons, contrasting results were obtained using quantitative approaches. While *Neun* mRNA expression tended to increase in the treated cultures, the proportions of NeuN-positive to total cells appeared to be negatively impacted by the supplement. CultureOne^TM^ supplement use and timing of implementation should, therefore, be carefully considered by the user depending on the experimental goals. This observation highlights the delicate balance between optimization for inclusion of astrocytes in the cultures and the impact of the optimization on neuronal populations. In view of all the data, we consider that the use of CultureOne^TM^ supplementation as a means of attenuating the contribution of glial cells, and astrocytes in particular, is advantageous and has a limited impact on neurons.

### 4.3. Applications and Perspectives

Finally, we provided evidence for the utility of these cultures in advancing neuroscience studies. Indeed, the protocol was effortlessly applied to a reporter transgenic mouse line, allowing the induction of the Cre recombinase in vitro. Cre activity was indeed successfully highlighted through the Ai14 reporter mouse line, therefore demonstrating the potential of this protocol for translating already existing in vivo models to the in vitro approaches.

Therefore, we propose this model as a strong basis for cellular neuroscience research focusing on pontine and medullary populations, which allows for a vast range of experimental procedures from pharmacological treatment, genetic modifications, immunodetection, and electrophysiological approaches.

## Figures and Tables

**Table 1:** Genotyping primers and protocols for transgenic mouse lines used in this study.

	Primers (5′-3′)			PCR Program		
Ai14 Rosa26R-tdTomato			1	95 °C	5 min	
	Fw	ggcattaaagcagcgtatcc	2	95 °C	30 s	35 cycles
			3	50 °C	1 min	
	Rv	ctgttcctgtacggcatgg	4	72 °C	40 s	
			5	72 °C	7 min	
CMV-CreER^T2^			1	95 °C	5 min	
	Fw	gtccgggctgccacgaccaa	2	95 °C	1 min	35 cycles
			3	65 °C	1 min	
	Rv	acggaaatccatgcgtcgaccagtt	4	72 °C	30 s	
			5	72 °C	7 min	

**Table 2:** References and targets of the primary antibodies used in this study.

Antigen	(Sub-) Cellular Target	Species	Catalog Number	Batch Number	Dilution	RRID
Bassoon	Presynapse	Mouse	75-491	475-3JS-69	1/500	AB_2716712
CHAT	Cholinergic neurons	Mouse	MAB305	2455649	1/100	AB_94647
GAD67	GABAergic neurons	Mouse	MAB5406	2042787	1/100	AB_2278725
Gephyrin	Inhibitory synapse	Rabbit	147 008	1-12	1/500	AB_2619834
GFAP	Astrocytes	Mouse	Sc-58766	J1308	1/500	AB_783554
Homer1	Excitatory synapse	Rabbit	160 003	3-72	1/500	AB_887730
MAP2	Neuronal body and dendrites	Chicken	PA1-16751	WC3231134A	1/2000	AB_2138189
NEUN	Neuronal nucleus	Mouse	MAB377	3018822	1/500	AB_2298772
OLIG2	Oligodendrocyte nucleus	Mouse	MABN50	2026416	1/100	AB_10807410
TH	Dopaminergic neurons	Mouse	MAB318	LV1541610	1/100	AB_2313764
VGLUT2	Glutamatergic neurons	Mouse	Ab79157	Gr99208-2	1/100	AB_1603114
IBA1	Microglia	Rabbit	019-19741	SAJ2266	1/500	AB_839504
Serotonin	Serotonergic neurons	Rabbit	20080	542021	1/500	AB_10718516

**Table 3:** References of the secondary antibodies used in this study.

Target Species	Alexa Fluor	Species	Catalog Number	Batch Number	Dilution	RRID
Anti-chicken	488	Goat	A11039	1937504	1/500	AB_2534096
Anti-chicken	647	Donkey	703-605-155		1/500	AB_2340379
Anti-mouse	488	Donkey	A21202	714258	1/500	AB_141607
Anti-mouse	555	Goat	4409	19	1/500	AB_1904022
Anti-rabbit	555	Goat	4413	20	1/500	AB_10694110

**Table 4:** Primers used for the gene expression study in RT-qPCR.

Target	Forward Primer (5′-3′)	Reverse Primer (5′-3′)
*Neun*	cttatggagcggtcgtgtatca	taactgtcactgtaggctgct
*Gfap*	catcgagatcgccacctaca	ctggaggttggagaaagtctgt
*Olig2*	cctggtgtctagtcgcccat	gacacagtccctcctgtgaa
*Hoxa5*	gcgcaagctgcacattagt	ggcatgagctatttcgatcc
*Th*	ctcctcagttctgtgcgtcg	gtcagagaagcccggatgg
*S100beta*	tggttgccctcattgatgtct	cccatccccatcttcgtcc
*Iba1*	cagggatttgcagggaggaaa	agtttggacggcagatcctc
*Vglut2*	tgaaatcagcaaggttggca	cccccgataggcacaatgat
*Gad67*	gagacaccctgaagtacggg	atgagaacaaacacgggtgc
*Chat*	ccattgtgaagcggtttggg	gccaggcggttgtttagataca
*Glyt2*	cacgctggagcacaacaatac	cagttccctcgggccttatt
*Gata3*	ttgataaggggccggttctg	cgggttcggatgtaagtcga
*Fezf2*	aaattatccatacccaggaaaaacc	ctgtgggtgagcttgtgattc
*Ppia*	aggattcatgtgccagggtg	ccgccagtgccattatgg
*H2a*	gctggtggtggtgtcatcc	tttcttcccgatcagcgatt
*36B4*	tgagattcgggatatgctgttg	ttccaatggtgcctctggaga
*Hprt*	gcttgctggtgaaaaggacctctcgaag	ccctgaagtactcattatagtcaagggcat
*Tbp*	acccttcaccaatgactcctatg	atgatgactgcagcaaatcgc
*Rpl32*	ggcaccagtcagaccgatat	caggatctggcccttgaac

## Data Availability

Data are contained within the article and Supplementary Materials.

## Supplementary Materials

The following supporting information can be downloaded at: https://www.mdpi.com/article/10.3390/cells14110758/s1, Figure S1: Macrodissection process for the isolation of E17.5 hindbrains.

## Abbreviations

The following abbreviations are used in this manuscript:

4-OHT	4-hydroxy-tamoxifen
Cx	Cortex
DIV	days in vitro
E	Embryonic day
EtOH	Ethanol
FBS	Fetal bovine serum
GABA	Gamma-aminobutyric acid
Hb	Hindbrain
HBSS	Hank’s balanced salt solution
PBS	Phosphate-buffered saline
PN	Postnatal day
PCR	Polymerase chain reaction
PFA	Paraformaldehyde
PLL	Poly-L-Lysine
RGC	Retinal ganglion cell
RT	Room temperature
RT-qPCR	Real-time quantitative polymerase chain reaction
TB	Tris-buffered saline
TBS-Tx	Tris-buffered saline with Triton X-100
