# Supplementary figures and images for: Primary Cell Cultures in Neurobiology: Optimized Protocol for Culture of Mouse Fetal Hindbrain Neurons
Source: Cells. 2025 May 22;14(11):758. doi: 10.3390/cells14110758 (PMC12153581; doi:10.3390/cells14110758)

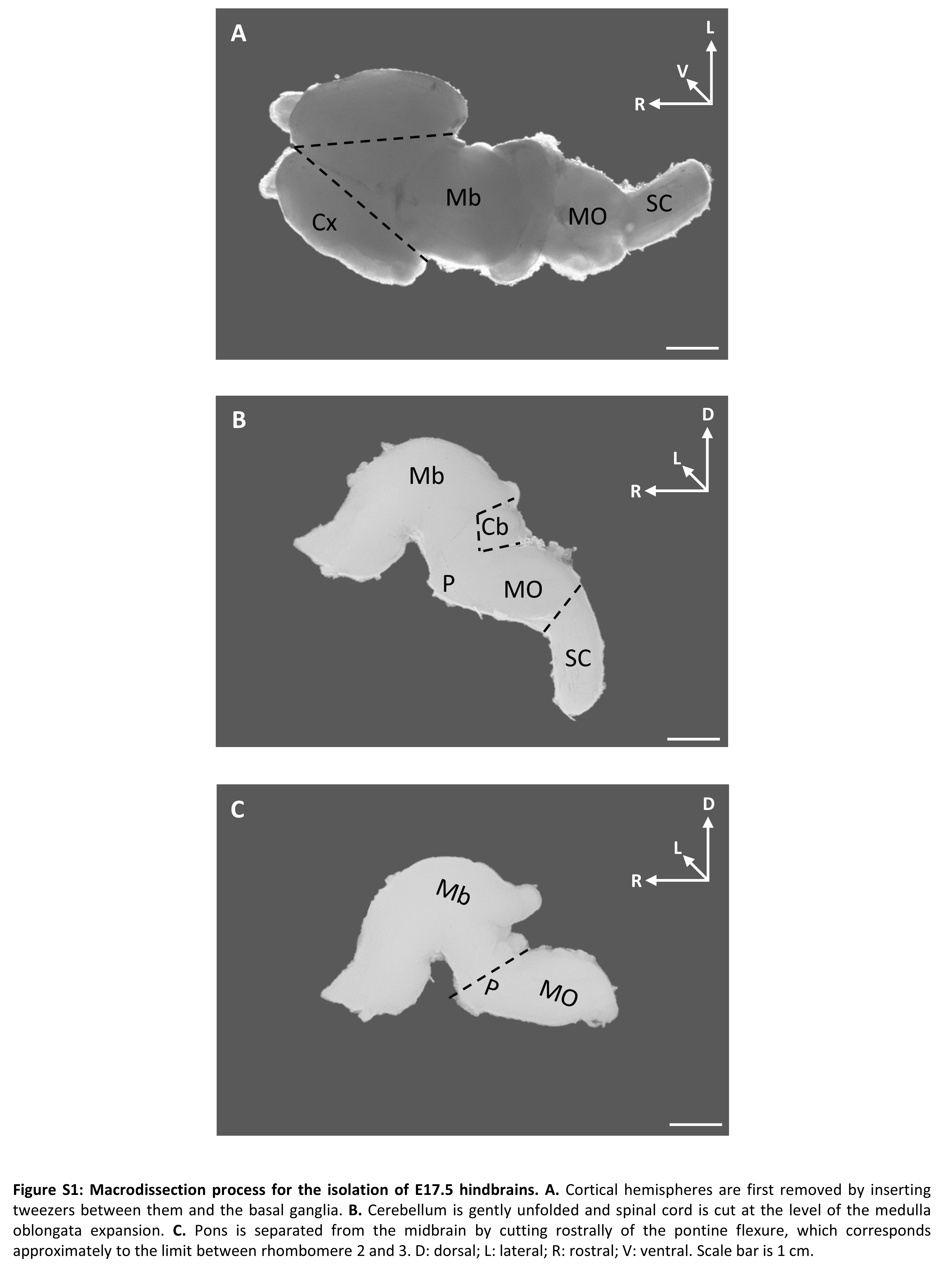

Supplement: Supplementary file 1 [file cells-14-00758-s001.zip › cells-3618133-supplementary.png]
